# A roadmap for interlayer excitons

**DOI:** 10.1038/s41377-021-00544-3

**Published:** 2021-05-08

**Authors:** Kai-Qiang Lin

**Affiliations:** Department of Physics, University of Regensburg, Regensburg, Germany

**Keywords:** Optical materials and structures, Electronics, photonics and device physics

## Abstract

Interlayer excitons in van der Waals heterostructures have tunable electron–hole separation in both real space and momentum space, enabling unprecedented control over excitonic properties to be exploited in a wide array of future applications ranging from exciton condensation to valleytronic and optoelectronic devices.

Light can trigger the separation of electrons and holes in semiconductors. Governed by the Coulomb force, electrons and holes can form bound quasiparticle states called excitons. Excitons often dominate the optical properties of semiconductors and are highly relevant for their optoelectronic applications, even up to room temperature when the binding energy exceeds the thermal energy. The duration of such a bound state of an electron and a hole, limited by annihilation and dissociation, is fundamentally determined by both how strongly they bond together and what distance lies between them. Tailoring this distance has been a formidable task in bulk semiconductors but recently became rather straightforward with the emergence of interlayer excitons in stacked van der Waals semiconductors, most notably transition metal dichalcogenide heterostructures.

By bringing different transition metal dichalcogenide monolayers together, an interface can be engineered in a so-called type-II band alignment (Fig. 1a, bottom panel)—electrons in the conduction band and holes in the valence band have energetic minima on opposite sides of the interface. When photoexcitation occurs, excitons are initially generated in the individual layers. Competing with the ultrafast intralayer radiative recombination, electrons and holes then tunnel to the respective lower-energy side within a timescale of 200 fs^1,2^, and interlayer excitons subsequently form (Fig. 1b). Owing to the reduced overlap between electron and hole wavefunctions in such interlayer excitons, they can have extraordinarily long lifetimes of up to a microsecond^3^, which allows for lateral diffusion before radiative recombination occurs (Fig. 1c). Being separated in the layered heterostructure by a van der Waals gap, the distance between electrons and holes is convenient to engineer.

**Fig. 1:**
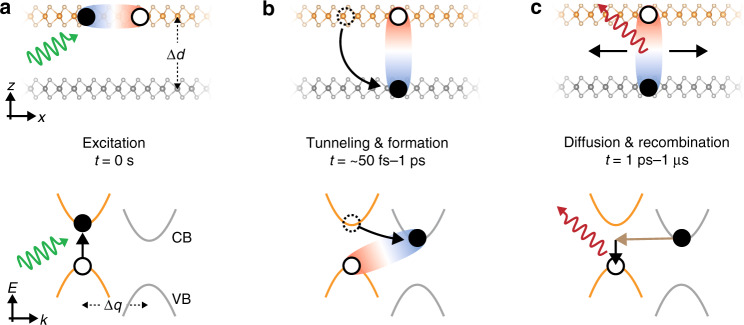
Illustration of interlayer exciton formation and relaxation in transition metal dichalcogenide heterobilayers. The top row illustrates the real-space side view of the heterobilayer separated by a distance *d*, and the bottom row shows the momentum-space type-II band alignment with the K valleys separated by a momentum Δ*q*. **a** Photoexcitation (green arrow) generates intralayer excitons. **b** Electrons or holes tunnel into the opposite layer with a lower potential energy, and interlayer excitons form. **c** Interlayer excitons diffuse, recombine, and emit light (red arrow). Momentum conservation can be met by the contribution of additional momentum (brown arrow) from other quasiparticles such as phonons or by localization of the exciton on a defect. The interlayer distance Δ*d* can be regulated by inserting thin insulators or by applying pressure, while the distance Δ*q* in momentum space can be engineered by twisting

The broad interest of the scientific community in spatially indirect excitons dates back to traditional quantum-well systems. The long lifetimes of these states combined with their permanent dipoles enabled a number of promising research avenues, including extensive exploration of excitonic Bose–Einstein condensation^4^ as well as excitonic circuitry^5^. The strength of the Coulomb interaction, however, was considerably weak, and the requirement of a small lattice mismatch in epitaxial growth presented a substantial constraint regarding the material combinations that can be explored in quantum wells. New possibilities have now arisen with van der Waals heterostructures, where the chosen semiconductor layers not only exhibit unusually robust excitons with a binding energy of several hundred meV but also can be mechanically stacked on top of each other with no need for lattice matching. Based on the very large variety of van der Waals materials identified^6^, many layer combinations can be formulated to support interlayer excitons with energies spanning a wide range.

To control the electron–hole distance of interlayer excitons in van der Waals heterostructures, atomically thin insulators such as hexagonal boron nitride (hBN) can be placed in between the semiconductor layers to regulate the interlayer spacing simply by the insulator thickness. In addition to this, the distance can also be conveniently tuned by external forces and fields. For instance, it was very recently shown that sufficient hydrostatic pressure applied to van der Waals heterostructures can continuously alter the interlayer distance and change the properties of the interlayer excitons^7^. When an out-of-plane electric field is applied to a van der Waals heterostructure, the electrons and holes are pulled in opposite directions, and the emission energy of the interlayer exciton can also be directly tuned through the quantum-confined Stark effect^8^. This strategy is particularly effective for realizing excitonic devices, where the spatial movement of the otherwise neutral excitons can nevertheless be controlled via electric fields^9^.

Control over the electron–hole distance arises not only in real space but also in momentum space. By implementing a twist between adjacent layers, the constituent electrons and holes of interlayer excitons can be separated in their momenta across the Brillouin zone^1^. Radiative recombination of interlayer excitons in this case requires additional momentum, which can be provided by other quasiparticles such as phonons (Fig. 1c, bottom panel); otherwise, the requirement can be mitigated following the uncertainty principle by exciton localization on a lattice defect. The radiative lifetime of the interlayer exciton can therefore be widely tuned by the twist angle. More interestingly, with a suitable combination of twist angle and lattice mismatch, a moiré superlattice can form between the two semiconductor layers^10^. When the moiré period is sufficiently large, a well-defined periodic potential can trap the interlayer excitons and induce correlations. The feature of such a potential modulation appears in not only excitonic transition energies^11–13^ but also exciton diffusion dynamics^14,15^.

Through careful design of the electron–hole separation in both real space and momentum space, the properties of interlayer excitons can be tailored for different applications. As an example, Wang et al. recently designed devices consisting of angle-aligned MoSe_2_–WSe_2_ double layers spaced by two or three layers of hBN and observed exciting signatures of interlayer exciton condensation up to a temperature of 100 K^16^. The control over the electron–hole separation is just one aspect though of the broad range of physics that needs to be addressed for interlayer excitons. Driven by a wide range of potential applications, including optoelectronic and valleytronic devices, excitonic circuits, Bose–Einstein condensation, excitonic superfluidity, and moiré-induced quantum emitter arrays, the field of interlayer excitons is developing and evolving rapidly^1,8,10^. Tremendous efforts by many researchers over the past years have expanded the field significantly into a plethora of new and exciting directions. In the article recently published in *Light: Science & Applications*, the pioneers of interlayer exciton research, Anlian Pan and colleagues, put together a roadmap for interlayer excitons in transition metal dichalcogenide van der Waals heterostructures, comprehensively covering most important aspects of interlayer excitons, including their formation mechanisms, relaxation and transport dynamics, and potential applications^17^. This timely review will provide valuable guidance to researchers in the field and be a great asset to the community.
